# Ergebnisse der filtrierenden Trabekulotomie (FTO) im Vergleich zur konventionellen Trabekulektomie (TE) – eine gematchte Fall-Kontroll-Studie

**DOI:** 10.1007/s00347-021-01365-w

**Published:** 2021-03-29

**Authors:** Caroline Maria Glatzel, Ágnes Patzkó, Juliane Matlach, Franz Grehn

**Affiliations:** 1 Universitätsklinik für Dermatologie und Venerologie, Josef Schneider Str. 11, 97080 Würzburg, Deutschland; 2 Universitäts-Augenklinik Würzburg, Josef Schneider Str. 11, 97080 Würzburg, Deutschland; 3 Augenklinik, Universitätsmedizin Mainz, Langenbeckstr. 1, 55131 Mainz, Deutschland

**Keywords:** Glaukomchirurgie, Filtrierende Glaukomchirurgie, Nicht-penetrierende Glaukomchirurgie, Augendrucksenkung, Matching, Glaucoma surgery, Filtering glaucoma surgery, Non-penetrating glaucoma surgery, Lowering of intraocular pressure, Matching

## Abstract

**Ziel:**

Ziel dieser Studie war es, die 2‑Jahres-Ergebnisse der filtrierenden Trabekulotomie (FTO) im Vergleich zur konventionellen Trabekulektomie (TE) bei primärem Offenwinkelglaukom, Pseudoexfoliationsglaukom und Pigmentglaukom zu untersuchen.

**Patienten und Methoden:**

Es wurden 30 konsekutive Patienten nach FTO und 87 Patienten nach TE nach intraokularem Druck (IOD) und Alter im Verhältnis 1:3 gematcht. Primärer Endpunkt war das Erreichen des Zieldrucks nach 2 Jahren. Als vollständiger Erfolg wurde ein IOD ohne Medikamente von ≤ 18 mm Hg bei gleichzeitiger IOD-Reduktion um ≥ 30 % definiert, als qualifizierter Erfolg, wenn hierfür zusätzlich Medikamente erforderlich waren. Sekundäre Endpunkte waren mittlere Drucksenkung, resultierende Sehschärfe, Komplikationen und nachfolgende Operationen. Die Operationstechnik der filtrierenden Trabekulotomie ist als Video zu diesem Beitrag abrufbar.

**Ergebnisse:**

Zwei-Jahres-Daten konnten von 27 Patienten aus der FTO-Gruppe und 68 Patienten aus der TE-Gruppe erhoben werden. Die Patienten beider Gruppen wurden vor Beginn der Studie bezüglich Alter und IOD gematcht, waren aber auch bezüglich Sehschärfe, Geschlecht und Medikation nicht unterschiedlich. Der Median des präoperativen IOD unter Therapie betrug in beiden Gruppen 23,0 mm Hg. Nach den oben genannten Kriterien wurde ein qualifizierter 2‑Jahres-Erfolg bei 70,4 % der FTO-Gruppe und bei 77,6 % der TE-Gruppe erzielt (*p* = 0,60), ein vollständiger 2‑Jahres-Erfolg bei 33,3 % der FTO-Gruppe und bei 56,7 % der TE-Gruppe (*p* = 0,07). Beide Operationsmethoden senkten den Augeninnendruck nach 24 Monaten signifikant (*p* < 0,001), und zwar auf 12,8 mm Hg in der FTO-Gruppe und 11,0 mm Hg in der TE-Gruppe. Die Sehschärfe war postoperativ bei beiden Gruppen etwas verringert, unterschied sich jedoch nicht signifikant zwischen beiden Gruppen. Komplikations- und Reoperationsrate waren gering und unterschieden sich nicht zwischen den Gruppen.

**Schlussfolgerung:**

FTO und TE sind nach 2 Jahren weitgehend gleichwertig bezüglich Zieldruck, IOD-Senkung, Sehschärfe und Komplikationen.

**Video online:**

Die Online-Version dieses Beitrags (10.1007/s00347-021-01365-w) enthält ein Video zur Operationstechnik der filtrierenden Trabekulotomie. Beitrag und Video stehen Ihnen auf www.springermedizin.de zur Verfügung. Bitte geben Sie dort den Beitragstitel in die Suche ein, das Zusatzmaterial finden Sie beim Beitrag unter „Ergänzende Inhalte“.

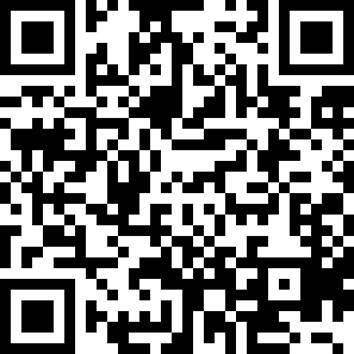

## Abstract (en)

**Objective:**

The aim of this study was to examine the 2‑year results of filtering trabeculotomy (FTO) compared to conventional trabeculectomy (TE) in primary open-angle glaucoma, pseudoexfoliation glaucoma, and pigmentary glaucoma.

**Patients and methods:**

Thirty consecutive patients after FTO and 87 patients after TE were included in the study. Both groups were matched 1:3 according to age and intraocular pressure (IOP). The primary endpoint was reaching the target IOP after 2 years. An IOP without medication of ≤ 18 mm Hg and an IOP reduction of ≥ 30% were defined as complete success, and as qualified success with medication. Secondary endpoints were mean IOP reduction, resulting visual acuity, complications and subsequent operations. The surgical technique of the FTO is available as a video for this article.

**Results:**

The 2‑year data from 27 patients with FTO and 68 patients with TE were evaluated. The patients in both groups were matched according to age and IOP but were also homogeneous with respect to visual acuity, gender, and medication. The preoperative IOP with glaucoma medication was 23.0 mm Hg in both groups. According to the defined criteria, a qualified 2‑year success was achieved in 70.4% of the FTO group and in 77.6% of the TE group (*p* = 0.60) and a complete 2‑year success in 33.3% of the FTO group and 56.7% of the TE group (*p* = 0.07). The IOP was significantly reduced after 24 months in both surgical groups (*p* < 0.001) and was 12.8 mm Hg in the FTO group and 11.0 mm Hg in the TE group. Visual acuity was moderately reduced postoperatively but did not differ significantly between the two groups. Complication and reoperation rates were low and not different between both groups.

**Conclusion:**

The results of FTO and TE are largely similar after 2 years in terms of complete and qualified success rate, lowering of IOP, visual acuity, and complications.

Filtrierende Glaukomoperationen senken nach wie vor den Augeninnendruck stärker als nicht penetrierende oder minimal invasive Glaukom (MIGS) Verfahren, erfordern aber eine aufwendige Nachbetreuung, um Misserfolge und Komplikationen zu vermeiden. In dieser Arbeit werden die 2‑Jahres-Ergebnisse einer filtrierenden Trabekulotomie im gematchten Vergleich zur Trabekulektomie dargestellt. Dieses Verfahren ist gleich wirksam und stellt eine Alternative zur Trabekulektomie dar.

Das Glaukom ist nach der Katarakt weltweit die zweithäufigste Erblindungsursache, jedoch die häufigste Ursache irreversibler Erblindung [33]. Nach einer Hochrechnung von Quigley et al. wird die beidseitige Erblindung durch Glaukom im Jahr 2020 weltweit auf 11,2 Mio. Menschen [22] und die Prävalenz des Glaukoms 2040 auf 111,2 Mio. Menschen [31] geschätzt. Aufgrund des demografischen Wandels ist der Anstieg der Glaukomprävalenz eine ernsthafte Herausforderung für die nähere Zukunft [5, 32].

Eine filtrierende Glaukomoperation senkt den intraokularen Druck (IOD) beim Glaukom sehr wirksam. Sie ist immer dann erforderlich, wenn sich Medikamente, Laser oder minimal-invasive Verfahren (MIGS) als unzureichend erwiesen haben [16, 19, 20, 30]. Seit 50 Jahren ist die Trabekulektomie eine Hauptstütze der primären Glaukomchirurgie [3, 15], aber ihre relativ hohe Komplikationsrate und der Nachbetreuungsaufwand geben immer wieder Anlass, Techniken mit gleicher Wirksamkeit, aber geringeren Nebenwirkungen anzustreben [1, 6, 8, 12–14, 17, 23–25]. Die hier analysierte neue Operationstechnik „filtrierende Trabekulotomie“ (FTO) wurde mit diesem Ziel entwickelt. Das Prinzip der Operationsmethode basiert wie bei der Trabekulektomie auf subkonjunktivaler Filtration. Im Gegensatz zur Trabekulektomie ist der künstliche Abflussweg jedoch prinzipiell anders, da durch die seitliche Lage der Trabekulotomien die Vorderkammer an der Präparationsstelle geschlossen bleibt. Daher tritt kein schwallartiger Abfluss von Kammerwasser auf. Der Abfluss wird bereits durch die Ostien des Schlemm-Kanals und danach durch den Skleradeckel gedrosselt. Eine Iridektomie ist deshalb nicht erforderlich. Das chirurgische Konzept der FTO zielt auch auf eine diffusere Verteilung des Kammerwassers unter der Bindehaut.

Die vorliegende Studie analysiert die 2‑Jahres-Ergebnisse dieser Operationsmethode. Die 1‑Jahres-Ergebnisse wurden bereits zuvor publiziert [18].

## Material und Methode

### Studiendesign

Das Design dieser Fall-Kontroll-Studie wurde von der Ethikkommission der Medizinischen Fakultät der Universität Würzburg überprüft und positiv begutachtet (Nr. 298/14); 117 primäre Glaukomoperationen durch denselben Operateur (FG) wurden eingeschlossen, und zwar 30 konsekutive Patienten, bei denen eine filtrierende Trabekulotomie (FTO) ausgeführt wurde, und 87 Patienten als gematchte Kontrollgruppe, bei denen eine Trabekulektomie (TE) in den 4 Jahren davor erfolgt war. Um die Vergleichbarkeit zwischen FTO-Gruppe und Kontrollgruppe (TE) zu erhöhen, wurden die Patienten beider Gruppen zuvor im Verhältnis 1:3 nach Alter und Augeninnendruck gematcht. Die Patienten waren aber auch in Bezug auf Sehschärfe, Geschlechtsverteilung, Verteilung der Glaukomformen POWG und PEX (TE-Gruppe 19,5 % PEX-Glaukome; FTO-Gruppe 20,0 % PEX-Glaukome) sowie bezüglich präoperativer Augenmedikation statistisch nicht signifikant unterschiedlich (Tab. 1). Follow-up-Daten für die Zeitpunkte 1,5 und 2 Jahre nach der jeweiligen Glaukomoperation wurden entweder an der Augenklinik der Universität Würzburg oder von den privaten Augenärzten mithilfe eines speziell entwickelten Fragebogens erhoben. Frühere Zeitpunkte entsprechen den Daten von Matlach et al. [18].

**Table 1:** 

Vor Operation	FTO	TE	Signifikanz *p*
Alter (Jahre)	67,0 ± 10,0	66,9 ± 9,2	0,94
Geschlecht (m – %)	36,7	49,4	0,29
Augen (RA – %)	50,0	52,9	0,83
POWG (%)	66,7	77,0	0,33
PEX-Glaukom (%)	20,0	21,8	1,00
Pigmentglaukom (%)	13,3	1,1	0,02
Pseudophakie (%)	30,0	10,3	0,02
Medikamente (%) (1/2/3/4 Wirkstoffklassen)	3,3/20,0/33,3/43,3	11,5/25,3/37,9/24,1	Jeweils *p* > 0,05
IOD – alle Patienten (mm Hg)	23,0	23,0	0,86
IOD nur PEX (mm Hg)	21,0	25,0	0,32
Visus Gesamtgruppe (logMAR (dezimal))	0,05 (0,9)	0,10 (0,8)	0,56
Visus PEX-Gruppe (logMAR (dezimal))	0,19 (0,63)	0,00 (1,0)	0,39

*IOD* intraokularer Druck, *FTO* filtrierende Trabekulotomie, *PEX* Pseudoexfoliationsglaukom, *POWG* primäres Offenwinkelglaukom, *RA* rechtes Auge, *TE* konventionelle Trabekulotomie

### Einschluss- und Ausschlusskriterien

Es wurden Patienten mit primärem oder spezifischem sekundärem Offenwinkelglaukom (Pseudoexfoliationsglaukom [PEX] und Pigmentglaukom [PG]) eingeschlossen. Patienten, die präoperativ systemische Medikamente zur Senkung des IOD erhielten, wurden nur eingeschlossen, wenn diese 3 oder mehr Tage vor der Operation abgesetzt wurden. Weitere Ausschlusskriterien waren ein primärer oder sekundärer Winkelverschluss, Normaldruckglaukom, angeborenes Glaukom, Neovaskularisationsglaukom (z. B. bei Diabetes mellitus oder nach Zentralvenenverschluss), absolutes Glaukom oder frühere inzisionelle Augenoperationen (z. B. kombinierte Phako-Trabekulektomie oder Netzhautoperationen) sowie Patienten mit mehr als 2 zykloablativen Eingriffen. Typ-2-Diabetiker ohne okuläre Manifestationen waren kein Ausschlusskriterium.

### Endpunkte

Als primärer Endpunkt wurde der 2‑Jahres-Erfolg, definiert als ein IOD von ≤ 18 mm Hg bei gleichzeitiger IOD-Reduktion um mindestens 30 % im Vergleich zum präoperativen IOD, gewertet. Als „vollständiger Erfolg“ wurde gezählt, wenn diese IOD-Reduktion ohne Glaukommedikation erreicht wurde, als „qualifizierter Erfolg“, wenn für dieses Erfolgskriterium eine medikamentöse Zusatztherapie erforderlich war [29]. Als sekundäre Endpunkte wurden die mittlere Drucksenkung über 2 Jahre, die IOD-Entwicklung im Verlauf (für einzelne Patienten und im Gruppenvergleich), die Sehschärfe sowie Komplikationen bzw. Re-Operationen ausgewertet. Eine statistische Subgruppenanalyse erfolgte für Augeninnendruck und Sehschärfe der PEX-Glaukome, nicht aber für die Pigmentglaukome, deren Anteil hierfür zu klein war. Da evtl. Komplikationen meist früh nach der Operation auftraten, sind hier nur solche aus dem späteren Verlauf (>1 Jahr) berücksichtigt.

### Operationstechniken

#### Filtrierende Trabekulotomie (FTO).

Nach üblicher Präparation eines Fornix-basalen Bindehautlappens wurde Mitomycin C in 4 Schwämmchen (2 × 8 mm; Flüssigkeitsaufnahme ca. 120 µl; Konzentration 0,2 mg/ml; 3 min) unter die Bindehaut und Tenon appliziert und danach mit 30 ml BSS gespült. Der äußere Skleradeckel wurde in halber Skleradicke in einer Größe von 4 × 4 mm bis in die klare Hornhaut angelegt. Danach wurde ein zweiter, etwas kleinerer zungenförmiger tiefer Skleradeckel in einer Ebene direkt über dem Ziliarkörper präpariert, wodurch der Schlemm-Kanal lokalisiert und in gesamter Breite eröffnet werden konnte. Dabei wurde die Innenwand des Kanals erhalten und die Ostien des Schlemm-Kanals auf beiden Seiten freigelegt. Der tiefe Skleradeckel wurde abgetrennt. Die Ostien des Schlemm-Kanals wurden identifiziert und der Schlemm-Kanal mit Trabekulotomiesonden nach Mackensen (Geuder Ophthalmic Instruments, Heidelberg, Deutschland) kanüliert. Durch Einschwenken in die Vorderkammer mit Drehpunkt am Deckelrand wird der Kanal auf beiden Seiten lediglich temporal bzw. nasal des Dissektionsbereichs eröffnet, während die freigelegte Innenwand des Schlemm-Kanals (Trabekel-Descemet-Membran) intakt bleibt. Daher ist eine Iridektomie nicht erforderlich. Abschließend wurde der äußere Skleradeckel mit 2 oder 4 einzelnen 10/0-Nylonfäden locker verschlossen. Wegen der Flussreduktion durch die Ostien des Schlemm-Kanals wurden diese Nähte lockerer als bei der konventionellen Trabekulektomie geknüpft. Die Bindehaut wurde durch eine fortlaufende 10/0-Nylon Matratzennaht [21] verschlossen. Die Operationstechnik ist als Video zu diesem Beitrag abrufbar.

#### Trabekulektomie (TE).

Bei der TE wurde der Skleradeckel mit 4 × 3,5 mm etwas kleiner dimensioniert. Mitomycin C wurde, wie oben beschrieben, angewendet. Zum Öffnen der Vorderkammer wurde ein 0,8 × 2 mm großes Fenster mit 2/3 Fläche in der klaren Hornhaut ausgeschnitten (Trabekulektomie) und eine periphere Iridektomie angelegt. Der Skleradeckel wurde mit 2 oder 4 10/0-Nylonnähten verschlossen, deren Spannung je nach Abflussleichtigkeit (BSS-Injektion in die Vorderkammer) justiert wurde.

### Statistische Methoden

Die statistische Analyse wurde mit SPSS (Version 23, IBM, Ehningen, Deutschland) und SAS Macro (Version 9.3, IBM, Ehningen, Deutschland) durchgeführt. *p*-Werte von ≤ 0,05 wurden als statistisch signifikant angesehen. Die 117 rekrutierten Patienten wurden auf der Grundlage ihres Alters (Bereich ±8 Jahre) und ihres präoperativen IOD (Bereich ±3 mm Hg) im Verhältnis 1:3 in eine Fallgruppe und eine Kontrollgruppe eingeteilt. Die Fallgruppe (FTO) bestand zu Beginn der Studie aus 30 Patienten, von denen 27 Patienten in der vorliegenden Studie 2 Jahre lang beobachtet werden konnten. Zu Beginn der Studie bestand die Kontrollgruppe (TE) aus 87 Patienten, von denen nach 2 Jahren Daten von 68 Patienten erhoben werden konnten. Dichotome Variablen wurden mit dem Fisher-Exact-Test auf Signifikanz geprüft, kontinuierliche Variablen wie Augeninnendruck oder Sehschärfe wurden mit dem Shapiro-Wilk-Test getestet. Ungepaarte, nicht normalverteilte Stichproben (z. B. Variabilitäten unter Patienten) wurden unter Verwendung des Mann-Whitney-U-Tests und ungepaarte normalverteilte Proben mit dem ungepaarten Students’ t‑Test getestet. Nicht normalverteilte Variablen unter gepaarten Proben (IOD-Entwicklung eines Patienten) wurden mit dem Wilcoxon-Vorzeichen-Rang-Test analysiert. Wenn beide Variablen normal verteilt waren, wurde der gepaarte Students’ t‑Test verwendet. Zur Analyse der Dauer des postoperativen Erfolgs (Überlebenszeit der beiden jeweiligen Operationen bezüglich vollständigen oder qualifizierten Erfolgs) wurde die kumulative Kaplan-Meier-Kurve berechnet. Zur besseren Anschaulichkeit wurden die Daten entsprechend den Guidelines der World Glaucoma Association auch in einem Streudiagramm (intraindividueller Vergleich) und als Boxplots (Augeninnendruckverlauf) dargestellt [29]. Boxplot-Diagramme zeigen den Median sowie die 25 %- und die 75 %-Perzentile, T‑Balken die 10 %- und 90 %-Perzentilen an. Daten außerhalb dieser Perzentilen wurden als zusätzliche Punkte in der Grafik angezeigt. Für normalverteilte Stichproben wurde ein Mittelwert mit Standardabweichung berechnet, andernfalls wurden der Median und der Interquartilbereich (IQR) verwendet.

## Ergebnisse

### Vergleichbarkeit der Studiengruppen.

Der Median des präoperativen Augeninnendrucks beider Studiengruppen war gleich und betrug 23,0 mm Hg (IQR 20,00–27,00) [18], da die Fälle gematcht waren. Die durchschnittliche Zahl der präoperativ gegebenen lokalen Glaukommedikamente war in beiden Gruppen nicht signifikant unterschiedlich (Tab. 1).

Nach 2 Jahren konnten 27 Patienten der FTO-Gruppe und 68 Patienten der TE-Gruppe nachuntersucht werden. Die Patienten beider Gruppen waren hinsichtlich Alter (*p*_präop_ = 0,94) und Geschlecht (*p*_präop_ = 0,29) vergleichbar. Vor der Operation umfasste die FTO-Gruppe signifikant mehr pseudophake Augen als die TE-Gruppe (*p* = 0,02), nach 2 Jahren war der Unterschied nicht mehr signifikant (*p* = 0,15). Zirka drei Viertel aller Patienten hatten ein primäres Offenwinkelglaukom (POWG_präop_ = 74,4 %, POWG_2a_ = 70,5 %), ca. ein Fünftel ein PEX-Glaukom (PEX_präop_ = 21,4 %, PEX_2a_ =24,2 %) und ca. 1/20 ein Pigmentglaukom (PG_präop_ = 4,3 %, PG_2a_ = 5,3 %). Die Verteilung des PEX-Glaukoms war in der FTO- bzw. TE-Gruppe innerhalb statistischer Grenzen gleich (Tab. 1). Die Zahl der Patienten mit Pigmentdispersionsglaukom war hingegen in der FTO-Gruppe signifikant höher als in der TE-Gruppe (*P*_präop_ = 0,02, *p*_2a_ = 0,02). Die Zahl der pseudophaken Augen war präoperativ in der FTO-Gruppe höher, nach 2 Jahren jedoch nicht mehr signifikant unterschiedlich (Tab. 2), d. h. der Zuwachs an Kataraktoperationen war nach TE höher als nach FTO.

**Table 2:** 

	FTO	TE	Signifikanz *p*
**1,5 Jahre postoperativ**			
Vollständiger Erfolg (%)	42,9	52,1	0,50
Qualifizierter Erfolg (%)	57,1	71,8	0,23
Medikamente (%) (1/2/3/4 Wirkstoffklassen)	0,0/17,9/10,7/3,6	4,2/9,9/14,1/7,0	Jeweils *p* > 0,05
IOD gesamt (mm Hg)	13,4	12,4	0,18
IOD PEX (mm Hg)	15,5 (*n* = 6)	11,0 (*n* = 15)	0,02
**2 Jahre postoperativ**			
Vollständiger Erfolg (%)	33,3	56,7	0,07
Qualifizierter Erfolg (%)	70,4	77,6	0,60
Medikamente (%) (1/2/3/4 Wirkstoffklassen)	11,1/14,8/11,1/11,1	4,3/10,1/8,7/5,8	–
IOD gesamt (mm Hg)	12,8	11,0	0,12
IOD PEX (mm Hg)	16,0 (*n* = 6)	11,0 (*n* = 17)	0,05
Visus Gesamtgruppe (logMAR (dezimal))	0,20 (0,63)	0,15 (0,7)	*p* > 0,05
Visus PEX-Gruppe (logMAR (dezimal))	0,05 (0,9)	0,13 (0,7)	*p* > 0,05

*IOD* intraokularer Druck, *FTO* filtrierende Trabekulotomie, *PEX* Pseudoexfoliationsglaukom, *TE* konventionelle Trabekulektomie

### Erfolgsrate der Augendrucksenkung.

**Figure 1:**
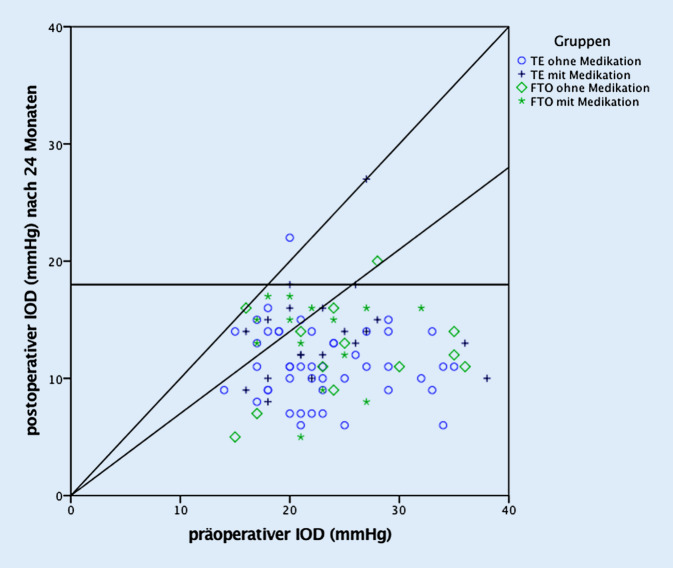

Ein Jahr nach der Operation wurde ein *vollständiger Erfolg* in beiden Operationsgruppen bei ca. 80 % der Patienten (FTO = 79,3 %; TE = 83,1 %) gefunden [18]. Nach 1,5 Jahren betrug der vollständige Erfolg 42,9 % in der FTO- und 52,1 % in der TE-Gruppe und nach 2 Jahren 33,3 % in der FTO- und 56,7 % in der TE-Gruppe. Der *qualifizierte Erfolg* betrug nach 1 Jahr 86,2 % in der FTO- und 83,1 % in der TE-Gruppe [18]. Nach 1,5 Jahren sank der qualifizierte Erfolg in beiden Gruppen ab (FTO = 57,1 %; TE = 71,8 %) und betrug 2 Jahre nach der Operation 70,4 % in der FTO-Gruppe und 77,6 % in der TE-Gruppe. Zwischen den Gruppen unterschieden sich vollständiger und qualifizierter Erfolg zu keinem Zeitpunkt signifikant (vollständiger Erfolg: *p*_12Mon_ = 0,78; *p*_18Mon_ = 0,50; *p*_24Mon_ = 0,07. Qualifizierter Erfolg: *p*_12Mon_ = 1,00; *p*_18Mon_ = 0,23; *p*_24Mon_ = 0,60), d. h. die Abnahme der Druckregulation beider Verfahren, war während der gesamten Beobachtungszeit weitgehend gleich (Tab. 2). Die 2‑Jahres-Ergebnisse des vollständigen und des qualifizierten Erfolgs aller Patienten sind im Streudiagramm von Abb. 1 dargestellt und lassen die Drucksenkung jedes einzelnen Auges nachvollziehen. Die postoperative Medikation ist in Tab. 2 aufgeführt.

### Kaplan-Meier-Analyse.

**Figure 2:**
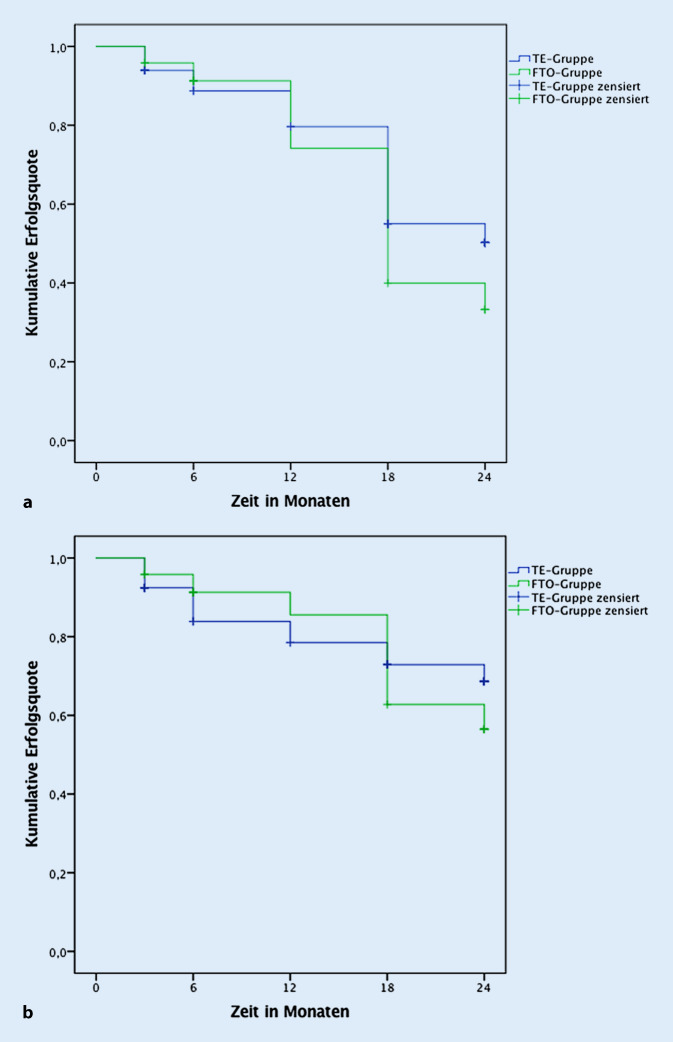

Im ersten Jahr war der *vollständige Erfolg* in der FTO-Gruppe höher als in der TE-Gruppe, im zweiten Jahr war dagegen der vollständige Erfolg in der TE-Gruppe höher. Basierend auf der Kaplan-Meier-Überlebenskurve, betrug die Wahrscheinlichkeit eines vollständigen Erfolgs nach 1,5 Jahren in der TE-Gruppe 55,0 % und in der FTO-Gruppe 39,9 %, nach 2 Jahren in der TE-Gruppe 50,2 % und in der FTO-Gruppe 33,3 % (Abb. 2a). Ein *qualifizierter Erfolg* wurde in der Kaplan-Meier-Überlebenskurve nach 1,5 Jahren in 72,9 % (TE) und 62,7 % (FTO), nach 2 Jahren in 68,6 % (TE) und 56,5 % (FTO) erreicht (Abb. 2b).

### Verlauf des Augeninnendrucks nach 1,5 bzw. 2 Jahren.

**Figure 3:**
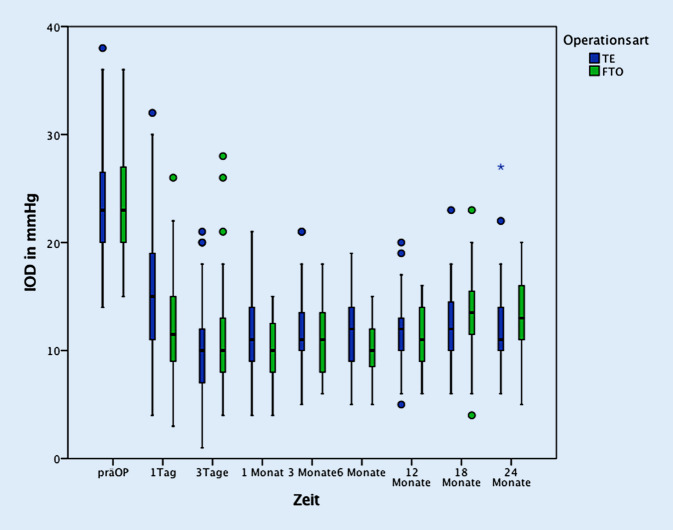

Nach 1,5 Jahren betrug der IOD (ggf. unter zusätzlicher Therapie) in der FTO-Gruppe 13,4 ± 4,2 mm Hg, in der TE-Gruppe 12,4 ± 3,2 mm Hg, nach 2 Jahren in der FTO-Gruppe 12,8 ± 3,8 mm Hg und in der TE-Gruppe 11,0 mm Hg (IQR 9,0–13,0). Nach 1,5 bzw. 2 Jahren unterschied sich der IOD zwischen diesen Gruppen nicht signifikant (*p*_18Mon_ = 0,18, *p*_24Mon_ = 0,12) (Abb. 3).

Ohne Berücksichtigung einer prozentualen Drucksenkung, d. h. bei starrer Obergrenze (vgl. Methode), war der 2‑Jahres-Augeninnendruck (±Medikamente) bei 97,0 % der TE-Gruppe und bei 100 % der FTO-Gruppe unter 21 mm Hg, bei 94,1 % der TE-Patienten und bei 96,3 % der FTO-Patienten unter 18 mm Hg, und bei 80,9 % der TE-Patienten und bei 59,3 % FTO-Patienten unter 15 mm Hg (vgl. Abb. 1). Alle postoperativen intraokularen Druckwerte waren im Vergleich zum präoperativen IOD signifikant erniedrigt (Abb. 3) sowohl in den gepoolten Werten aller Operationen als auch in den beiden Gruppen (FTO und TE) getrennt.

### Sehschärfe.

**Figure 4:**
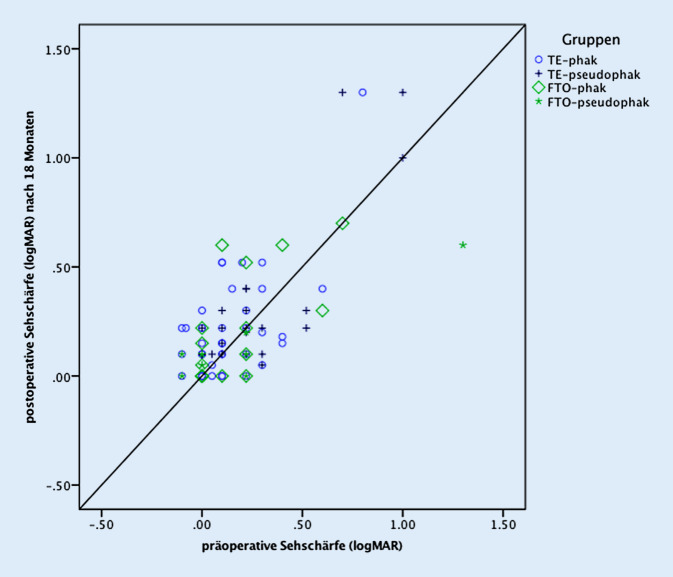

Die korrigierte Sehschärfe für die Ferne (SF) betrug vor Operation in der TE-Gruppe 0,10 logMAR (dezimal 0,8) und in der FTO-Gruppe 0,05 logMAR (dezimal 0,9; *p* = 0,6) [18] und 2 Jahre postoperativ bei TE 0,15 logMAR (dezimal 0,7) und bei FTO 0,20 logMAR (dezimal 0,63). Die postoperative SF war in beiden Gruppen im Vergleich zum präoperativen SF-Wert signifikant reduziert (*p* < 0,05). Die SF unterschied sich jedoch zwischen beiden Operationstechniken nicht signifikant (*p* ≥ 0,5) (Tab. 2). Das Streudiagramm von Abb. 4 zeigt den individuellen Sehschärfenvergleich aller Augen vor Operation und nach 2 Jahren.

### Pseudoexfoliationsglaukom (Subgruppenanalyse) und Pseudophakie.

Der postoperative Augeninnendruck (mit und ohne Medikamente) von Patienten mit PEX-Glaukom war nach Operation signifikant niedriger als präoperativ (*p* < 0,001). Die Unterschiede zwischen FTO und TE waren zwar grenzwertig signifikant, die Zahl der FTO Operationen jedoch sehr klein (Tab. 2). Die postoperative Sehschärfe war während des gesamten Follow-up sowohl bei phaken als auch bei pseudophaken PEX-Augen nicht signifikant verringert (Tab. 2). Der Zuwachs an Kataraktoperationen innerhalb der 2 Jahre war nach TE höher (14,0 %-Punkte) als nach FTO (9,9 %-Punkte) (Tab. 1 und 2). Die Anzahl der Patienten mit* Pigmentglaukom* war zu gering, um eine separate Bewertung zu ermöglichen (Tab. 2).

### Postoperative Komplikationen.

Mögliche Komplikationen wie langfristige Hypotonie mit flacher Vorderkammer, hypotensive Makulopathie, Aderhautschwellung sowie externe Sickerkissenfistel, Blebitis oder Endophthalmitis wurden bei beiden Operationsmethoden nach 1,5 und 2 Jahren nicht beobachtet. Kein Patient beider Gruppen zeigte nach 1,5 bzw. 2 Jahren einen Augeninnendruck von ≤ 5 mm Hg.

### Reoperationen.

In der FTO-Gruppe wurden ein Sickerkissen-Needling (nach 1,5 Jahren) und eine Suturolyse (nach 2 Jahren) sowie eine YAG-Laser-Goniopunktur durchgeführt. In der TE-Gruppe wurden bei 2 Augen innerhalb von 2 Jahren eine ALT und bei 1 Auge ein Sickerkissen-Needling durchgeführt (Tab. 2).

## Diskussion

Seit ihrer Erstbeschreibung durch Cairns im Jahr 1968 [3] erwies sich die Trabekulektomie über 50 Jahre lang als die effektivste augendrucksenkende Operation. Allerdings sind Nachbehandlung und Beherrschung von Komplikationen anspruchsvoll. Durch die Entwicklung der nicht penetrierenden Glaukomchirurgie [23, 24], Verwendung moderner Schlauchimplantate [4] und Einführung minimal-invasiver Glaukomeingriffe (MIGS) [2, 11, 26–28] hat sich das operative Spektrum in den letzten 10 Jahren stark erweitert, sodass jetzt die Operationswahl stärker auf die individuelle Situation des Patienten fokussiert werden kann. Nach wie vor sind aber die sog. Filtrationseingriffe, also die Ableitung des Kammerwassers unter die Bindehaut ohne Schlauchsysteme bei Primäreingriffen stärker und dauerhafter drucksenkend als alle anderen genannten Verfahren [9, 10, 24].

Die vorliegende Studie vergleicht eine neue Operationsmethode, die filtrierende Trabekulotomie (FTO) mit der Trabekulektomie (TE) unter der Zielsetzung, das Nebenwirkungsspektrum zu verbessern, ohne den drucksenkenden Effekt des Eingriffs zu reduzieren. Die hier vorgelegten 1,5- und 2‑Jahres-Ergebnisse ergänzen die bereits publizierten 1‑Jahres-Daten [18].

Im Gegensatz zu den meisten anderen Studien, die als Erfolgskriterium Grenzwerte von 21 oder 18 mm Hg festlegen, wurde entsprechend den Vorschlägen der WGA und EGS [7, 29] eine zusätzliche prozentuale Augendrucksenkung (hier 30 %) zugrunde gelegt, was immer dann von Bedeutung ist, wenn die Augeninnendruckwerte bereits vor der Operation nicht sehr stark erhöht sind. Andernfalls würden auch Operationen als Erfolg klassifiziert, die individuell keine oder nur eine geringe Augendrucksenkung erreicht haben. Das Streudiagramm von Abb. 1 erlaubt es aber, auch andere Zieldruckkriterien für die hier vorgestellten Daten anzuwenden.

Das Prinzip der Operationsmethode „filtrierende Trabekulotomie“ (FTO) besteht darin, die Vorderkammer an der Operationsstelle nicht zu eröffnen (anders als bei der Trabekulektomie), sondern wie bei der tiefen Sklerektomie [25] eine Descemet-Trabekel-Membran im Operationsgebiet zu erhalten, sodass kein Irisprolaps erfolgt und keine Iridektomie erforderlich wird. Die Filtration wird erreicht, indem der benachbarte Schlemm-Kanal nach beiden Seiten mit einer Sondentrabekulotomie eröffnet wird, sodass Kammerwasser über die Ostien des Schlemm-Kanals in den Sklerasee unter den Skleradeckel fließen kann und danach unter die Bindehaut gelangt, wo es ähnlich der TE resorbiert wird (s. Video). Da bei dieser Methode bewusst eine Filtration angestrebt wird, erfolgt wie bei der TE eine intraoperative Applikation von MMC. Der Kammerwasserfluss wird bei der FTO zunächst durch Ostien des Schlemm-Kanals reguliert, die das Kammerwasser in den Sklerasee leiten. Der zweite Widerstand wird durch den Skleradeckel erzeugt, von wo das Kammerwasser in den subkonjunktivalen Raum gelangt. Im Gegensatz dazu wird bei TE der Kammerwasserfluss allein durch einen einzigen Widerstand, nämlich am Skleradeckel kontrolliert, hauptsächlich durch die Anzahl und Spannung der Nähte. Durch die 2‑stufige Kontrolle des Abflusses bei der FTO sind die Sickerkissen diffuser verteilt und weniger dünn-zystisch als bei TE. Während 1 Jahr nach Operation beide Methoden eine gleiche Drucksenkung erbringen [18], ist nach 2 Jahren, gemessen am „vollständigen Erfolg“, die FTO etwas weniger wirksam als die TE, wenn auch nicht statistisch signifikant (Abb. 2a und 3; Tab. 2).

Insgesamt wurden im Beobachtungszeitraum von 2 Jahren keine signifikanten Unterschiede in der Drucksenkung beider Methoden gefunden. Betrachtet man jedoch den Gesamtverlauf aller Untersuchungszeitpunkte im 1. und 2. Jahr, dann lässt sich im 2. Jahr eine (nicht signifikante) Tendenz zu besserer Drucksenkung nach Trabekulektomie (TE) erkennen (Abb. 3). Andererseits ist dieser neue Filtrationseingriff (FTO) wahrscheinlich stärker wirksam als nicht-penetrierende Verfahren (tiefe Sklerektomie, Kanaloplastik), wenn man die randomisierten Vergleichsstudien nicht-penetrierender Verfahren gegenüber der Trabekulektomie zugrunde legt [17, 23, 24].

Auch bezüglich Visuserhalt wurden in unserer Studie keine signifikanten Unterschiede zur Trabekulektomie gefunden, auch nach Berücksichtigung der unterschiedlichen Zahl pseudophaker Augen in beiden Gruppen. Die Subgruppenanalyse der Augen mit Pseudoexfoliation zeigte trotz höherer Ausgangsdruckwerte eine gleich effektive Augeninnendrucksenkung, und auch im Visusverlauf konnte kein Unterschied zu Augen mit primärem Offenwinkelglaukom gefunden werden (Tab. 2). Bei keinem der Patienten traten schwerwiegende Komplikationen auf, weder in der frühpostoperativen Phase [18] noch langfristig. Nach 2 Jahren hatte die FTO die gleiche Komplikationsrate wie die TE (Tab. 2).

Durch das Matchingverfahren im Verhältnis 1:3 wird ein möglicher Bias zwischen beiden Gruppen stärker reduziert als bei 1:1-Matching*. Die Schwere des Glaukomschadens wurde nicht gesondert ins Studienprotokoll aufgenommen, da der ursprüngliche Ansatz, die Gesichtsfeldprogression zu ermitteln, über einen Zeitraum von 1 bis 2 Jahren nicht aussichtsreich erschien. Ein Bias durch unterschiedliche Glaukomvorschädigung beider Gruppen erscheint jedoch wenig wahrscheinlich, da beide Operationsverfahren unter gleicher Indikationsstellung des Operateurs durchgeführt wurden (FTO ausschließlich anstelle von TE). Eine multivariate Analyse von verschiedenen Systemerkrankungen (z. B. Diabetes mellitus) wurde wegen der begrenzten Fallzahl nicht durchgeführt, insbesondere neovaskuläre und entzündliche Glaukome waren aber laut Studienprotokoll ausgeschlossen.

Trotz der Einschränkung durch den zwar gematchten, jedoch nicht randomisierten Vergleich und die limitierte Fallzahl zeigt diese Studie eine gute Wirksamkeit dieses neuen Filtrationseingriffes.

## Fazit

Die filtrierende Trabekulotomie (FTO) entspricht in Bezug auf Augeninnendruck, Sehschärfe und Erfolgsrate der Trabekulektomie (TE). Nach 2 Jahren war die Komplikationsrate in beiden Gruppen sehr gering und nicht signifikant unterschiedlich. Die FTO erwies sich als eine gute und sichere Alternative zur TE. Die vorliegende Studie rechtfertigt eine Weiterentwicklung dieser neuen Operationstechnik.

## Supplementary Information


